# Burnout and Insomnia Among Greek Physicians Affiliated with the Athens Medical Association After the Acute Phase of the COVID-19 Pandemic: Prevalence and Contributing Factors

**DOI:** 10.3390/epidemiologia7030073

**Published:** 2026-05-24

**Authors:** Dimosthenis Akrivakis, Dimitrios Lamprinos, Maria Patatoukou, Stavroula Alevizou, Georgios Zoumpoulis, Theodoros Pouletidis, Paraskevi Deligiorgi, Panagiotis Georgakopoulos, Evangelos Oikonomou, Gerasimos Siasos, Kostas A. Papavassiliou, Christos Damaskos, Georgios Rachiotis, Dimitrios Schizas, Georgios Marinos

**Affiliations:** 1 Medical School, National and Kapodistrian University of Athens, 11527 Athens, Greece; 2 Emergency Care Department, Laiko General Hospital, 11527 Athens, Greece; dimitrislamprinos@gmail.com (D.L.);; 3 General Hospital of Athens “G. Gennimatas”, 11527 Athens, Greece; panos.k.georgakopoulos@gmail.com; 4 First Department of Cardiology, Hippokration General Hospital, Medical School, National and Kapodistrian University of Athens, 11527 Athens, Greece; 5 Third Department of Cardiology, Thoracic Diseases General Hospital Sotiria, Medical School, National and Kapodistrian University of Athens, 11527 Athens, Greece; 6 First Department of Respiratory Medicine, “Sotiria” Hospital, Medical School, National and Kapodistrian University of Athens, 11527 Athens, Greece; 7 N.S. Christeas Laboratory of Experimental Surgery and Surgical Research, Medical School, National and Kapodistrian University of Athens, 11527 Athens, Greece; 8 Department of Hygiene and Epidemiology, Faculty of Medicine, University of Thessaly, 41334 Larissa, Greece; 9 First Department of Surgery, National and Kapodistrian University of Athens, Laikon General Hospital, 11527 Athens, Greece; 10 Department of Hygiene, Epidemiology and Medical Statistics, School of Medicine, National and Kapodistrian University of Athens, 11527 Athens, Greece

**Keywords:** COVID-19, physicians, burnout, insomnia, mental health

## Abstract

**Background:** The COVID-19 pandemic has been a global crisis, affecting healthcare systems and professionals worldwide. This study investigates the prevalence and factors associated with burnout and insomnia among Greek physicians affiliated with the Athens Medical Association after the acute phase of the COVID-19 pandemic. **Methods:** Data were collected through an anonymous online survey distributed to active physician members of the Athens Medical Association between 15 June 2023 and 15 July 2023. Burnout was assessed using the Maslach Burnout Inventory (MBI), and insomnia was assessed using the Athens Insomnia Scale (AIS). Descriptive, unadjusted, and multivariable analyses were performed. **Results:** A total of 1023 physicians participated. Insomnia (AIS ≥ 6) affected 83.0% of the participants. Based on standard MBI cut-offs, 52.4% had high emotional exhaustion, 35.9% had high depersonalization, and 39.2% had low personal accomplishment. In multivariable logistic regression, older age was significantly associated with lower odds of insomnia, while public-sector employment and high concern about future career consequences were associated with higher odds. In multiple linear regression models, a higher AIS total score was significantly associated with higher emotional exhaustion and depersonalization and with lower personal accomplishment. **Conclusions:** These findings suggest high rates of insomnia and burnout in this physician sample. Greater insomnia was significantly associated with less favorable scores across all three burnout dimensions. Younger age, public-sector employment, and higher concern about future career consequences were associated with insomnia. These findings should be interpreted as associations, rather than causal effects.

## 1. Introduction

The pandemic of COVID-19, which started with reports of pneumonia cases of unknown etiology in Wuhan, China, in December 2019, spread throughout the world in a matter of months and rapidly became the deadliest pandemic of the twenty-first century [1,2]. The World Health Organization (WHO) characterized it as a pandemic on 11 March 2020, and as of today, more than 700,000,000 cases have been reported, with almost 7,000,000 deaths worldwide [3,4]. During the pandemic, hospital ward admissions surged dramatically, and along with the overcrowded intensive care units, the health sector was under pressure to manage the crisis [5]. Healthcare workers (HCWs), especially physicians, who were on the frontline of the pandemic, put themselves at risk from COVID-19 [6]. Overwhelming workload, shortages of personal protective equipment, being positively tested for COVID-19, and worrying about infecting their loved ones were the main factors that caused not only physical but also mental exhaustion among physicians, making them vulnerable to emotional exhaustion [7].

Numerous studies around the globe documented the mental health challenges that physicians faced during that time, showing unprecedented stress and burnout, confirming that emotional tension can increase the risk of burnout [8]. Burnout (BO) over the last decade has become a major psychosocial problem with increasing prevalence among physicians, occurring when chronic stress in the workplace is left unmanaged (World Health Organization, 2019) [9]. The triad of energy exhaustion increased mental distance from one’s work (i.e., cynicism or negativism), and a sense of lack of accomplishments characterizes this psychological syndrome [10]. Physicians, a high-risk group for developing BO due to the challenging nature of the profession, were familiar with the term BO long before COVID-19, with multiple studies confirming that [11]. Physicians’ BO is a complex and multifaced issue impacting not only physicians’ health but also patient care and the healthcare system [12,13]. In terms of the physician’s health, both anxiety and depression have a positive correlation with BO [14]. In addition, physicians who are burned out are more likely to succumb to alcohol abuse and are at increased risk of suicidal ideation [14,15].

A relationship between insomnia (IS) and BO has also been suggested, with poor quality of sleep discovered among burned-out physicians [16]. IS was also prevalent among physicians long before the COVID-19 pandemic, with night shifts, long working hours, and stress, among other factors, contributing to poor sleep quality or quantity [17]. According to the fifth edition of the Diagnostic and Statistical Manual for Mental Disorders (DSM-5), IS is defined as dissatisfaction with sleep quantity or quality and is usually associated with one or more of the following: (1) difficulty initiating sleep, (2) difficulty maintaining sleep and (3) early-morning awakening with inability to return to sleep [18]. Even though the prevalence of IS and BO among physicians was already high, during the pandemic there seemed to be an increase in their occurrence, especially among those who worked in the COVID-19 clinics, with multiple studies supporting this [17,19,20,21].

The same dynamics that affected the rest of the world also applied to Greece, with Greek physicians suffering the same consequences as every other physician in the world due to the enormous pressure put on the healthcare sector [22]. In Greece, the first case of COVID-19 was reported on 26 February 2020, and since then, more than 6,000,000 cases and almost 38,000 deaths have been documented [23,24]. Greece encountered four major COVID-19 waves: the first from September to December 2020, the second from February to June 2021, the third from December 2021 to February 2022, and the fourth from June to September 2022 [24,25]. On 4 May 2023, the WHO declared that it is no longer regarded as “a public health emergency of international concern” [26]. Data for the present study were collected between June and July 2023, after the acute pandemic waves in Greece and after the World Health Organization declared that COVID-19 no longer constituted a public health emergency situation. In this later COVID-19 context, there has been no large-scale study of physicians in Greece examining burnout and insomnia. Therefore, the aim of this study was to determine the prevalence of burnout and insomnia and to identify associated demographic, occupational, and COVID-19-related factors in this physician population. We also examined the association between insomnia severity and the three MBI dimensions.

## 2. Materials and Methods

This study was based on an anonymous online survey, distributed to the members of the largest medical association in Greece (A.M.A). The data were collected over the period of 15 June 2023, to 15 July 2023, spanning a total of 30 days. The survey was conducted using Google Forms, ensuring anonymity by not requesting any personal information from participants. According to A.M.A. records, there are approximately 3000 active members, all of whom are required to provide an email address to maintain their membership. The questionnaire was distributed exclusively via email by the Athens Medical Association to its active physician members. Participation was restricted to recipients within the Association’s mailing list, and the survey did not circulate through public links or open recruitment channels. The inclusion criteria were active membership to the A.M.A., access to the Internet, and voluntary involvement. The population ensured a sample of healthcare professionals with diverse backgrounds and specialties. Participants were informed about the purpose of the study and were provided with a clear explanation of the survey’s components. The participants provided anonymous informed consent on the survey platform before they could proceed to the electronic completion of the questionnaire. The study followed the principles of the Helsinki Declaration of 1975, as revised in 2013. The protocol study was approved by the Board of the A.M.A (number: 2859/22 May 2023).

The survey consisted of multiple sections covering different aspects. Demographic information included questions on age, gender, and marital status. COVID-19-related questions assessed the specialty practiced, type of employment (National Health System-NHS, private, or self-employed), vaccination status including booster dose, history of COVID-19 infection among participants or their family members, concerns about contracting the virus, history of hospitalization due to COVID-19, and whether oxygen therapy or intubation was required during hospitalization. Work environment conditions were also explored, addressing concerns about transmitting COVID-19 to others, perceived risk of infection and burnout across different pandemic waves, material shortages in the workplace and their impact on work quality, satisfaction with rest after night shifts, and job security concerns over the past year. Additionally, the survey focused on emotional exhaustion and burnout, inquiring about concerns regarding income loss due to the pandemic and potential future career impacts.

The design of the questionnaire was based on established psychological assessment tools to measure burnout (BO) and insomnia (IS) accurately. Two primary scales were utilized: the Maslach Burnout Inventory (MBI) for assessing BO and the Athens Insomnia Scale (AIS) for evaluating IS levels. Previously validated Greek versions of the MBI and AIS were used [27,28].

### Statistical Analysis

Continuous variables are presented as means and standard deviations (SD), while categorical variables are presented as frequencies and percentages. No missing data were observed in the analyzed variables.

The questionnaire included two established instruments. Burnout was assessed using the Maslach Burnout Inventory (MBI), which consists of 22 items providing three subscales: emotional exhaustion (9 items; possible score range 0–54), depersonalization (5 items; possible score range 0–30), and personal accomplishment (8 items; possible score range 0–48). Each item is scored from 0 to 6. For descriptive purposes, standard cut-offs were used to classify emotional exhaustion as low (≤20), medium (21–30), or high (≥31), depersonalization as low (≤5), medium (6–10), or high (≥11), and personal accomplishment as high (≥42), medium (36–41), or low (≤35); lower personal accomplishment scores indicate less favorable outcomes. Insomnia was assessed using the Athens Insomnia Scale (AIS), which includes 8 items scored from 0 to 3, providing a total score range of 0–24; a total score of 6 or higher is commonly used as the cut-off for insomnia screening positivity [27,28]. Internal consistency of the study scales was evaluated using Cronbach’s alpha. In the present study population, Cronbach’s alpha was 0.912 for emotional exhaustion, 0.782 for depersonalization, 0.778 for personal accomplishment, and 0.840 for AIS.

The AIS total score was analyzed both as a continuous variable and as a binary variable, using the established cut-off of 6 points or greater to indicate positive screening of insomnia. MBI subscales were additionally categorized according to standard cut-offs into low, medium, and high levels.

For the multivariable analyses, some categorical variables were recoded into dichotomous variables to improve interpretability. Specifically, marital status was recoded into married/cohabiting vs. other, employment sector into public vs. private, economic concern into none/little vs. much/very much, and concern about future career consequences after the pandemic into low vs. high concern.

Group differences in categorical variables were assessed using Pearson’s chi-square test. As the distributions of MBI subscales and AIS deviated from normality, comparisons of MBI subscale scores between participants with AIS scores below 6 points and those with AIS scores of 6 points and above were performed using the Mann–Whitney U test. Associations between AIS total score and MBI subscale scores were further examined using Spearman’s rank correlation coefficient.

To search for possible factors significantly associated with positive insomnia screening (AIS ≥ 6), multivariable binary logistic regression analysis was performed. Age was entered as a continuous variable. Other variables included gender, specialty, self/family COVID-19 diagnosis, fear of COVID-19 infection, fear of transmitting COVID-19, marital status (married/cohabiting vs. other), employment sector (public vs. private), economic concern (none/little vs. much/very much), and concern about future career consequences after the pandemic (low vs. high). Results are presented as odds ratios (ORs) with 95% confidence intervals (CIs). Because insomnia screening positivity was highly prevalent in the study sample, the odds ratios may overestimate the corresponding prevalence ratios and should therefore be interpreted cautiously as measures of association.

To examine factors significantly associated with burnout, three separate multiple linear regression models were made with emotional exhaustion, depersonalization, and personal accomplishment as dependent variables. AIS total score and the same variables used in the logistic regression model were entered. Regression coefficients (B), standardized coefficients (β), 95% CIs, and *p*-values are reported. SPSS 23.0 was used for the analyses and statistical significance was set at *p* < 0.05.

Covariates for the multivariable models were selected a priori based on clinical relevance, study objectives, and the availability of demographic, occupational, and COVID-19-related variables that may act as confounders.

## 3. Results

The questionnaire was sent to all the members of the Athens Medical Association (A.M.A.) via email, and 1023 answered (response rate approximately 34%), with no missing data in the analyzed variables. Out of the 1023 participants, 54.3% (n = 555) of them were women, and 45.7% (n = 468) were men. The mean age of the participants was 50.12 years (SD 11.48). Most participants were married (61.6%), worked in a pathological specialty (57.5%), and were employed in the public sector (47.5% NHS, with additional respondents from university and army settings). Most of the responders (90.0%, n = 921) were vaccinated with the booster dose of the COVID-19 vaccine, underscoring a high vaccination rate among Greek physicians. Moreover, 89.0% of the participants (n = 916) had themselves or a family member diagnosed with COVID-19 infection. Over half of the responders (57.8%, n = 591) were worried about being infected or having a member of their family be diagnosed as positive for COVID-19, and 73.1% (n = 748) were afraid of transmitting COVID-19 to someone in their close environment. Only 1.5% (n = 15) of the infected physicians were hospitalized, and out of them, seven individuals required oxygen supplementation. Most of the participants reported that the highest possibility of getting infected with COVID-19 from work was during the second wave of the pandemic, from February to June 2021 (32.2%, n = 329, and 34.2%, n = 350, respectively). Almost one-third of the participants were afraid of the economic consequences of the pandemic, while the same percentage experienced the fear of future consequences for their careers after the end of the pandemic (Table 1).

The mean AIS total score was 10.81 (SD 5.16). The mean MBI subscale scores were 30.10 (SD 13.38) for emotional exhaustion, 8.79 (SD 7.09) for depersonalization, and 38.82 (SD 6.56) for personal accomplishment. Using the AIS cut-off (AIS ≥ 6), 83.0% of participants screened positive for insomnia. Based on standard MBI cut-offs, 52.4% of participants had high emotional exhaustion, 35.9% had high depersonalization, and 39.2% had low personal accomplishment (Table 2).

In unadjusted analyses, women had a higher prevalence of insomnia positive screening than men (86.3% versus 79.1%, *p* = 0.002). Insomnia was also more common among participants who reported fear of COVID-19 infection (86.0% versus 78.9%, *p* = 0.003), fear of transmitting COVID-19 (85.2% versus 77.1%, *p* = 0.002), and self or family COVID-19 diagnosis (84.2% versus 72.9%, *p* = 0.003) (Table 3).

Participants screening positive for insomnia (AIS ≥ 6) were significantly associated with higher depersonalization and emotional exhaustion scores and lower personal accomplishment scores than those with AIS scores below six. Specifically, participants with AIS ≥ 6 had higher mean ranks for depersonalization (549.18 versus 330.57, *p* < 0.001) and emotional exhaustion (569.57 versus 231.09, *p* < 0.001), whereas participants with AIS < 6 had higher mean ranks for personal accomplishment (733.74 versus 466.55, *p* < 0.001). Spearman correlation analysis showed that AIS total score was positively correlated with emotional exhaustion (*p* < 0.001) and depersonalization (*p* < 0.001) and negatively correlated with personal accomplishment (*p* < 0.001).

In multivariable logistic regression analysis, older age was significantly associated with lower odds of insomnia screening positivity (OR 0.955, 95% CI 0.938–0.972, *p* < 0.001). Public-sector employment was associated with higher odds of insomnia compared with private-sector employment (OR 1.598, 95% CI 1.107–2.306, *p* = 0.012). High concern about future career consequences after the pandemic was also associated with insomnia, with participants with low concern having lower odds of insomnia than those with high concern (OR 0.429, 95% CI 0.246–0.749, *p* = 0.003). No associations were observed for gender, specialty, self or family COVID-19 diagnosis, fear of infection, fear of transmitting COVID-19, marital status, or economic concern (Table 4).

Three multiple linear regression models were used to examine the association of insomnia with burnout. The model for emotional exhaustion explained 40.7% of the variance (adjusted R^2^ = 0.407, *p* < 0.001). A higher AIS total score was significantly associated with higher emotional exhaustion (B = 1.248, β = 0.482, *p* < 0.001). Older age was associated with lower emotional exhaustion (B = −0.206, β = −0.176, *p* < 0.001), while female gender (B = 2.636, β = 0.098, *p* < 0.001), self or family COVID-19 diagnosis (B = 3.153, β = 0.072, *p* = 0.003), and higher concern about future career consequences (B = 2.893, β = 0.096, *p* = 0.001) were associated with higher levels of emotional exhaustion. Employment sector showed a borderline association (B = −1.379, *p* = 0.050), suggesting lower emotional exhaustion in the private sector compared with the public sector.

The model for depersonalization explained 25.8% of the variance (adjusted R^2^ = 0.258, *p* < 0.001). A higher AIS total score was significantly associated with higher depersonalization (B = 0.320, β = 0.233, *p* < 0.001). Older age was associated with lower depersonalization (B = −0.187, β = −0.303, *p* < 0.001). Female gender was associated with lower depersonalization (B = −1.334, β = −0.094, *p* = 0.001), while greater concern about future career consequences was associated with higher depersonalization (B = 1.539, β = 0.097, *p* = 0.002). In addition, private-sector employment was associated with lower depersonalization compared with public-sector employment (B = −1.767, β = −0.124, *p* < 0.001).

The model for personal accomplishment explained 24.9% of the variance (adjusted R^2^ = 0.249, *p* < 0.001). A higher AIS total score was significantly associated with lower personal accomplishment (B = −0.442, β = −0.347, *p* < 0.001). Older age was associated with higher personal accomplishment (B = 0.128, β = 0.224, *p* < 0.001). Greater concern about future career consequences was associated with lower personal accomplishment (B = −1.305, β = −0.088, *p* = 0.006), whereas private-sector employment was associated with higher personal accomplishment compared with public-sector employment (B = 0.855, β = 0.065, *p* = 0.028) (Table 5).

**Figure 1:**
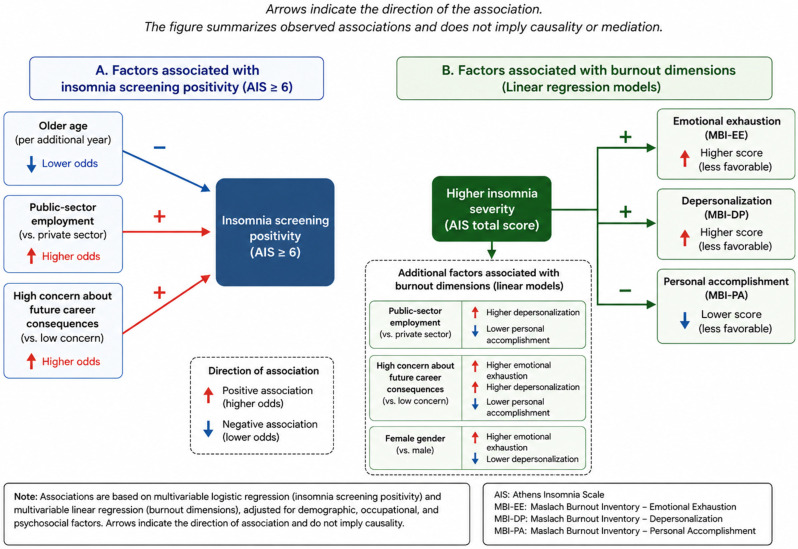
Summary of significant associations of the adjusted analyses.

To facilitate the interpretation of the adjusted findings, a summary figure was added to illustrate the direction of the observed associations. This figure is intended as a visual summary of the regression results and not as a causal model (Figure 1).

## 4. Discussion

The present study examined the presence and contributing factors of burnout (BO) syndrome and insomnia (IS) among Greek physicians after the acute phase of the COVID-19 pandemic, using a validated self-report instrument in a large cross-sectional sample. As of now, there is not yet a large-scale study investigating this. The main findings were that insomnia screening positivity was high, that all three burnout dimensions were significantly associated with insomnia, and that younger age, public-sector employment, and concern about future career consequences were significantly associated with insomnia in the adjusted analysis. In addition, a higher AIS total score remained independently associated with higher emotional exhaustion and depersonalization, as well as lower personal accomplishment across multivariable linear regression models. Notably, 90.0% of the participants had received the booster dose of the COVID-19 vaccine, a higher rate than reported in recent Greek studies [29]. This could be explained by both the rising number of positive cases and the availability of more compelling data on the effectiveness of booster doses, leading physicians to opt for vaccination [30].

Our results showed that the fear of being infected or having a family member diagnosed with COVID-19 was significant among our participants. Over half of the participants (57.8%) reported increased anxiety because of such fear, with 73.1% of the responders having a fear of transmitting the virus to someone in their close environment. Ultimately, 89.5% of them were infected or had a close one infected by COVID-19. Self or family COVID-19 diagnosis was associated with higher emotional exhaustion in the adjusted linear model, but not with insomnia in the final logistic regression model. This suggests that personal or family exposure to COVID-19 may be associated with emotional exhaustion more than with sleep disturbance. Although anxiety and fear are normal responses to threats such as a pandemic, it appears that physicians’ fear of infecting their family members, their fear of forced social isolation from family and loved ones, and their hazardous work environment resulted in increased anxiety and higher levels of BO [31]. A study of comparative significance in the U.S. revealed that over 80.0% (n = 2579) of the participants experienced interpersonal difficulties, such as fearful feelings for their families due to their possible COVID-19 exposure at work and moral distress about infecting family [32]. A notable finding in our study was that around one-third (33.0%) of the participants had a fear of economic consequences due to the pandemic, and a fear of future consequences for their careers when the pandemic came to its end. In the adjusted logistic model, higher concern about future career consequences was significantly associated with insomnia screening positivity. In the linear models, the same factor was associated with higher emotional exhaustion and depersonalization and with lower personal accomplishment. This suggests that concern about future career consequences may be associated with both sleep disturbance and burnout-related symptoms. A similar study investigating the effects of the pandemic on orthopedic and trauma surgeons of the two largest German professional associations reported that 77.41% of them reported themselves as having economic constraints [33].

Physicians work under immense pressure, striving to save lives on an everyday basis, and even before the COVID-19 pandemic, their mental health has been compromised, impacting their quality of life [34]. The COVID-19 outbreak caused healthcare professionals to experience heightened levels of anxiety and depression [35]. The decline in their psychological well-being has also been correlated to BO [34]. BO is one of the main reasons physicians struggle in their everyday lives outside of work, as it has been suggested that there is an association between BO anxiety, depression, and suicidality [34]. Another thing strongly related to BO that impacts the lives of healthcare workers is substance abuse, as alcohol dependence has been stated as a significant problem among physicians [35]. Taking all of these factors into consideration, and factoring in the onset of the COVID-19 pandemic, which exacerbated the prevalence of BO and further disrupted physicians’ ability to enjoy their personal lives, it becomes clear how significantly both elements have affected their everyday lives and, by extension, their overall quality of life.

Our collected data using the MBI scale indicate that a significant number of Greek physicians suffered from BO [27]. Women remained significantly associated with higher emotional exhaustion in the linear regression analysis. Interestingly, female gender was associated with lower depersonalization in the adjusted model. These findings suggest that gender may relate to burnout. BO’s effect on female physicians after the pandemic was significant, contributing remarkably to their emotional exhaustion and extreme emotional stress. Females are more likely to experience BO because of the strong influence of emotional exhaustion [36]. Findings from another study conducted among physicians in the Netherlands focusing on gender differences regarding BO showed that women’s BO seems to be triggered by emotional exhaustion [37]. A similar study reported that women were more likely to report BO and one of the factors that women reported that exacerbated that probability was childcare or caregiving responsibilities [38]. Numerous research papers show that women’s workload increased during the COVID-19 crisis compared to men’s, leading to an exacerbation of gender inequalities despite the progress being made in the last few decades [39]. For this reason, female physicians experience considerable burdens during and after the acute phase of the COVID-19 pandemic, particularly in the contexts of their response to stress, social isolation, work–life integration, and autonomy. Another factor that was correlated to emotional exhaustion besides the female gender was the history of positivity for COVID-19. Similarly, results from an Iranian study showed that the risks of psychological symptoms such as depression, anxiety, stress, and PTSD are significantly more common in healthcare workers with a history of COVID-19 infection in comparison to those who never got infected [40].

Furthermore, incorporating the AIS into our study revealed that IS was prevalent among Greek physicians during that time, with 83.0% of participants scoring six or above on the AIS. Although this figure should be interpreted cautiously because the AIS is a screening instrument and the study design is cross-sectional, it suggests a considerable burden in this physician population. Our results are consistent with those of other studies. In a study from Iran, the most prevalent mental health problem among study participants during the pandemic was IS (66.5%) [41]. It has been demonstrated in a sample of Bahrain healthcare workers during the COVID-19 outbreak that 61.0% (n = 257) of the participants had poor-quality sleep and moderate to severe stress [42]. The higher prevalence of poor-quality sleep may be related to concerns about the contagious nature of COVID-19. In the unadjusted analyses, insomnia was more frequent among women, among participants reporting fear of infection, among those worried about transmitting COVID-19, and among those reporting self or family COVID-19 diagnosis. However, these associations were not significant after adjustment for other variables. An association between younger age and insomnia was found, giving the explanation that younger physicians may experience greater professional insecurity. Compared with private-sector employment, public-sector employment was associated with higher odds of insomnia in the logistic regression model. In the linear models, public-sector employment was also associated with higher depersonalization and lower personal accomplishment, with a borderline association for emotional exhaustion. One possible explanation is that public-sector physicians may have been more exposed to increased workload demands, as they were the frontline physicians. However, because employment sector may also be associated with differences that are not measured, like different work schedules or different job roles, this association should be interpreted with caution. Multiple meta-analyses displayed that frontline healthcare workers had a higher prevalence of IS and sleep disturbances compared to the second-line ones [43].

In the unadjusted analyses, women showed a higher prevalence of insomnia screening positivity than men. However, this association did not remain statistically significant in the final logistic regression model. Though the prevalence of IS in women was notably higher than in men even before the COVID-19 outbreak, there appeared to be an exacerbation of the phenomenon during the pandemic [44]. A comparable study reported that female healthcare workers suffer more from IS than males [41]. Furthermore, a review of multiple meta-analyses revealed that the prevalence of sleep disturbances or IS was higher in females working in healthcare than in males [43]. Another important point is to consider the correlation between BO and IS. Our statistical analysis revealed a significant correlation between AIS scores above six and elevated BO levels across all MBI dimensions. A higher AIS total score was associated with higher emotional exhaustion and depersonalization and with lower personal accomplishment. Among the three, the strongest association was observed for emotional exhaustion, both in the correlation analysis and in the linear regression model. A recent study trying to identify risk factors for BO and IS characterized BO as “the strongest risk factor for insomnia” and found that IS is also a risk factor for BO [45]. Similarly, a study aiming to assess IS and sleep quality among physicians with BO indicated a clear relationship between IS and BO [16]. The same survey showed that the higher the BO score, the higher the sleep disturbances.

Despite their correlation and the fact that BO was and is an issue among healthcare workers, IS has and continues to affect them, and its impact beyond BO is profound. One of the reasons why IS is prevalent among healthcare workers is due to long working hours and night shifts [17]. A study evaluating permanent night workers in an overnight lab protocol linked IS to functional and cognitive impairments, showing how harmful such shifts can be on a long-term basis [46]. A systematic review notes that up to 30% of shift workers experience IS symptoms [47]. A nationwide survey showed that the prevalence of sleep aid use among emergency physicians was high, indicating that those physicians struggle with IS [17]. Among the sleep aids reported, the two most prevalent were alcohol and benzodiazepine, respectively [17]. An assessment of emergency physicians made before the COVID-19 crisis showed that poor sleep, along with perceived effectiveness, affects personal safety, as one-third of the physicians had fallen asleep while driving [48].

As mentioned before, BO was already a significant and multidimensional problem, as it affects the healthcare system, the patient’s outcomes [2], and the lives of healthcare workers [12,13,19]. Studies revealed that its occurrence not only remained high but also increased during the COVID-19 pandemic [49]. Post-pandemic data indicate that the prevalence of BO remains comparably high to pre-pandemic levels, with a notable increase in the number of physicians meeting the threshold for emotional exhaustion, nearly double the rate observed prior to the pandemic [50]. Another survey found that the prevalence of severe BO was even higher after the pandemic than before, 30.4% and 34.5%, respectively, showing that even after the COVID-19 crisis, one-third of healthcare workers are affected by it [49]. Therefore, even following the conclusion of the pandemic, BO continues to affect healthcare workers’ lives at rates that are either consistent with or exceed those observed prior to the pandemic.

It is, therefore, important to raise awareness about physicians’ BO. Targeted interventions both in the work environment and in the health behaviors of the individuals are needed, as both can have meaningful reductions in BO among healthcare workers [51]. Organizational-directed approaches such as rescheduling shifts, reducing workload, discussion meetings, or communication skill training have been shown to boost the reduction in BO [52]. A study exploring the implementation of workload intervention in primary care clinics showed that those who received such an implementation showed not only an improvement in the workload but also a decrease in the emotional exhaustion dimension of BO [53]. Individual-focused interventions, such as educational and mindfulness-based ones, should be actively promoted, as they have the potential to reduce both BO and occupational stress [54]. In conclusion, physicians’ BO remains an ongoing concern. Only with proactive interventions can we mitigate its impact and improve the quality of life for those affected. Otherwise, it will continue to influence individuals and societies alike in the foreseeable future.

The study provides valuable insights into the impact of the COVID-19 pandemic on physicians in Greece. However, it is essential to acknowledge certain limitations. Our sample of physicians from the A.M.A. may introduce some sampling bias and non-response bias. The physicians who participated who are also members of this association may differ in demographic and professional characteristics from non-members, limiting the universality of the results. Secondly, the study relies on self-reported data, which is susceptible to response bias. Physicians experiencing higher burnout or insomnia may have been either more likely to respond because of the salience of the topic or less likely to participate because of fatigue, workload, or limited availability. Although several covariates were considered in the analyses, residual confounding by unmeasured factors cannot be excluded. Furthermore, because insomnia screening positivity was highly prevalent in the sample, the odds ratios from the logistic regression model may overestimate the corresponding prevalence ratios and should be interpreted cautiously. Finally, data collection occurred after the most acute pandemic waves; therefore, the observed associations may be a combination of post-pandemic burden and work-related stress, rather than acute pandemic exposure alone.

## 5. Conclusions

In conclusion, insomnia and burnout were prevalent in this sample of physicians affiliated with the Athens Medical Association. Greater insomnia severity was significantly associated with higher emotional exhaustion and depersonalization and with lower personal accomplishment. Younger age, public-sector employment, and greater concern about future career consequences were significantly associated with insomnia screening positivity, while public-sector employment and future career concerns were also associated with less favorable burnout outcomes. Because of the cross-sectional design, these findings should be interpreted as associations, rather than causal effects. Nevertheless, they highlight the need for targeted interventions to support the well-being of health care professionals, particularly among younger physicians and those working in the public sector. Future studies are needed to investigate the relationship between insomnia and burnout and to determine whether these associations persist over time.

## Figures and Tables

**Table 1:** Demographic, occupational, and COVID-19-related characteristics of the participants.

Variable	n	%
Gender		
Male	468	45.7
Female	555	54.3
Marital status		
Single	225	22.0
Married	630	61.6
Divorced	93	9.1
Cohabit	64	6.3
Widower	11	1.1
Specialty		
Pathological specialty	588	57.5
Surgical specialty	250	24.4
Laboratory specialty	111	10.9
Other	74	7.2
Employment		
NHS doctor	486	47.5
Private sector-self employed	174	17.0
Private clinic	292	28.5
University doctor	22	2.2
Army doctor	49	4.8
Booster vaccination		
No	102	10.0
Yes	921	90.0
Self/family COVID-19 diagnosis		
No	107	10.5
Yes	916	89.5
Fear of infection		
No	432	42.2
Yes	591	57.8
Fear of transmission		
No	275	26.9
Yes	748	73.1
Job insecurity		
No	610	59.6
Yes	413	40.4
Economic concern		
No	245	23.9
Little	429	41.9
Much	258	25.2
Very much	91	8.9

**Table 2:** Scale characteristics, internal consistency, and categorical classification.

Measure	Mean ± SD	Cronbach’s Alpha	Category	n	%
AIS total score	10.81 ± 5.16	0.840	AIS < 6	174	17.0
			AIS ≥ 6	849	83.0
Emotional exhaustion	30.10 ± 13.38	0.912	Low	276	27.0
			Medium	211	20.6
			High	536	52.4
Depersonalization	8.79 ± 7.09	0.782	Low	417	40.8
			Medium	239	23.4
			High	367	35.9
Personal accomplishment	38.82 ± 6.56	0.778	Low	401	39.2
			Medium	358	35.0
			High	264	25.8

For personal accomplishment, lower scores indicate less favorable outcomes.

**Table 3:** Unadjusted analyses between selected variables and insomnia screening positivity (AIS ≥ 6).

Variable	AIS < 6 n (%)	AIS ≥ 6 n (%)	*p*-Value
Gender			0.002
Male	98 (20.9)	370 (79.1)	
Female	76 (13.7)	479 (86.3)	
Fear of infection			0.003
No	91 (21.1)	341 (78.9)	
Yes	83 (14.0)	508 (86.0)	
Fear of transmission			0.002
No	63 (22.9)	212 (77.1)	
Yes	111 (14.8)	637 (85.2)	
Self or family COVID-19 diagnosis			0.003
No	29 (27.1)	78 (72.9)	
Yes	145 (15.8)	771 (84.2)	

*p*-values from Pearson’s chi-square test.

**Table 4:** Logistic regression analysis for insomnia positive screening (AIS ≥ 6).

Factors	OR	95% CI	*p*-Value
Age (years)	0.955	0.938–0.972	<0.001
Female gender	0.725	0.504–1.044	0.084
Self/family COVID-19 diagnosis	0.663	0.404–1.090	0.105
Fear of infection	0.728	0.480–1.104	0.135
Fear of transmission	0.763	0.491–1.184	0.228
Married/cohabiting vs. other	0.726	0.493–1.068	0.104
Public vs. private employment	1.598	1.107–2.306	0.012
Much/very much economic concern vs. none/little	0.822	0.527–1.284	0.390
Low vs. high future career concern	0.429	0.246–0.749	0.003

OR = odds ratio; CI = confidence interval.

**Table 5:** Multiple linear regression analyses for emotional exhaustion, depersonalization, and personal accomplishment.

Factor	Emotional Exhaustion B (β), *p*-Value	Depersonalization B (β), *p*-Value	Personal Accomplishment B (β), *p*-Value
AIS total score	1.248 (0.482), *p* < 0.001	0.320 (0.233), *p* < 0.001	−0.442 (−0.347), *p* < 0.001
Age (years)	−0.206 (−0.176), *p* < 0.001	−0.187 (−0.303), *p* < 0.001	0.128 (0.224), *p* < 0.001
Female gender	2.636 (0.098), *p* < 0.001	−1.334 (−0.094), *p* = 0.001	−0.368 (−0.028), *p* = 0.318
Specialty	−0.310 (−0.022), *p* = 0.372	−0.321 (−0.042), *p* = 0.119	−0.351 (−0.050), *p* = 0.068
Self/family COVID-19 diagnosis	3.153 (0.072), *p* = 0.003	0.635 (0.027), *p* = 0.319	−0.201 (−0.009), *p* = 0.735
Fear of infection	−0.490 (−0.018), *p* = 0.530	0.203 (0.014), *p* = 0.661	0.148 (0.011), *p* = 0.731
Fear of transmission	1.242 (0.041), *p* = 0.153	0.144 (0.009), *p* = 0.779	0.280 (0.019), *p* = 0.559
Married/cohabiting vs. other	−0.462 (−0.016), *p* = 0.522	−0.459 (−0.030), *p* = 0.284	−0.021 (−0.001), *p* = 0.959
Employment sector (private vs. public)	−1.379 (−0.051), *p* = 0.050	−1.767 (−0.124), *p* < 0.001	0.855 (0.065), *p* = 0.028
Much/very much economic concern	−0.050 (−0.002), *p* = 0.949	−0.763 (−0.051), *p* = 0.099	0.700 (0.051), *p* = 0.104
High future career concern	2.893 (0.096), *p* = 0.001	1.539 (0.097), *p* = 0.002	−1.305 (−0.088), *p* = 0.006
Model fit	Emotional Exhaustion	Depersonalization	Personal Accomplishment
Adjusted R^2^	0.407	0.258	0.249

**B** = unstandardized regression coefficient; **β** = standardized regression coefficient.

## Data Availability

The study data are available from the corresponding author (G.M.) upon reasonable request.
